# Investigation of an outbreak of Elizabethkingia meningoseptica on a pediatric intensive care unit

**DOI:** 10.3205/dgkh000390

**Published:** 2021-05-31

**Authors:** Mehmet Erinmez, Ayse Büyüktas Manay, Yasemin Zer

**Affiliations:** 1 Department of Medical Microbiology, School of Medicine, Gaziantep University, Gaziantep, Turkey

**Keywords:** Elizabethkingia meningoseptica, pediatric intensive care unit, infection control intervention, nosocomial infections

## Abstract

**Objective:** This paper reports an *Elizabethkingia meningoseptica* outbreak on a pediatric intensive care unit with emphasis on investigation of outbreak source, infection control interventions, patient characteristics and comparative antimicrobial susceptibility results.

**Methods:** This was an ambidirectional cohort study conducted in a university hospital 20-bed pediatric intensive care unit. Patient ages ranged from 4 to 11 months, with a median age of 9 months. 83% of the patients had severe underlying conditions. Samples from staff and environmental surfaces were obtained to identify a common source of infection. Antimicrobial susceptibility tests of isolated bacteria were done using the disk diffusion method and the Vitek^®^2 automated system.

**Results:** Environmental surveillance revealed contamination of the water reservoirs of two different mechanical ventilators. *In-vitro* antimicrobial susceptibility testing results with two different methods (Vitek^®^2 and disk diffusion) were coherent for most of the investigated antibiotics, but without coherence for ciprofloxacin and levofloxacin. Resistance was found to the relatively new antibiotics ceftaroline and ceftazidime-avibactam.

**Conclusions:** *E. meningoseptica* is a significant cause of nosocomial infections, with high mortality especially in children. Investigation of the outbreak source and continuation of intensive infection control precautions are vital to handle *E. meningoseptica* outbreaks in PICUs. Using quinolones according to testing results of automated AST systems may lead to inadequate treatment and foster the selection of resistant strains.

## Zusammenfassung

**Zielsetzung:** Es wird über einen Ausbruch mit *Elizabethkingia meningoseptica* auf einer pädiatrischen Intensivstation (PICU), die Ausbruchsquelle, das Ausbruchsmanagements, die Patientencharakteristika und die antimikrobielle Empfindlichkeit des Erregers berichtet.

**Methode:** Es handelte sich um eine ambidirektionale Kohortenstudie auf einer pädiatrischen Intensivstation einer Universitätsklinik mit 20 Betten. Das Alter der Patienten betrug 4 bis 11 Monate mit einem mittleren Alter von 9 Monaten. 83% der Patienten hatten schwere Grunderkrankungen. Es wurden Proben vom Personal und von Umgebungsoberflächen genommen, um die Infektionsquelle zu identifizieren. Die antimikrobielle Empfindlichkeit der isolierten Bakterien wurde mittels Agardiffusionstst und parallel mittels Vitek^®^2 bestimmt.

**Ergebnisse:** Die Umgebungsüberwachung ergab eine Kontamination des Wasserreservoirs von zwei verschiedenen Beatmungsgeräten. Die Ergebnisse der Empfindlichkeitstestung mittels Agardiffusionstest und Vitek^®^2 stimmten für die meisten Antibiotika mit Ausnahme von Ciprofloxacin und Levofloxacin überein. Resistenzen waren gegen Ceftarolin und Ceftazidim-Avibactam nachweisbar.

**Schlussfolgerung:** *E. meningoseptica* kann nosokomiale Infektionen mit hoher Mortalität insbesondere bei Kindern verursachen. Die Identifizierung der Ausbruchsquelle und intensive Infektionskontrollmaßnahmen sind entscheidend für die Beherrschung von *E. meningoseptica* Ausbrüchen auf PICUs. Der Einsatz von Chinolonen kann zu inadäquater Behandlung führen und die Selektion resistenter Stämme begünstige

## Introduction

*Elizabethkingia meningoseptica (E. meningoseptica)* is a non-fermentative, nonmotile, oxidase-positive, nonspore-forming, Gram-negative aerobic bacillus, formerly named *Flavobacterium meningosepticum* and *Chryseobacterium meningosepticum* [1], [2], [3]. *E. meningoseptica* grows as yellow colonies on blood agar, like bacteria in the Chryseobacterium group. Infection with *E. meningoseptica* may be associated with endocarditis, cellulitis, wound infection, bacteremia, abscess, dialysis-related peritonitis and meningitis, and hospital-acquired pneumonia [4], [5].

Abundant in nature, *Elizabethkingia* species are found in soil and water reservoirs. Various studies have shown that *E. meningoseptica* may even be found in chlorine-treated water, often colonizing sink basins and taps and creating potential reservoirs for nosocomial infections [6], [7], and it has also been detected on hospital surfaces. *E. meningoseptica* is more virulent than other species in the same genus, and positive cultures of about half of adult patients and about two-thirds of neonatal patients show true infection [1].

Various devices and equipment – especially those that have fluid contact, e.g., respirators, syringes, and intubation tubes – may be contaminated and cause colonization of patients [7]. It is a serious infection agent, especially for newborns and immunocompromised patients who are intubated in the hospital [8]. *E. meningoseptica* infection due to contaminated central venous catheters (CVC) and prosthetic valves have been reported [9]. Person-to-person transmission is not expected, as manifested by the low rates of infection among neonates housed in adjacent bassinets [5]. There are contradictory reports about the antimicrobial susceptibility of *E. meningoseptica*, but combination regimens that include vancomycin are likely to be effective for patients with *E. meningoseptica* infections [10]. This study was conducted after subsequent positive clinical cultures of *E. meningoseptica* on a pediatric intensive care unit (PICU) at a university hospital. Antimicrobial susceptibilities, treatment regimens, and clinical outcomes were also evaluated to discuss clinical characteristics of pediatric patients, outbreak containment measures, and possible new treatment options. 

## Methods

### Medical Care Settings and Patients 

 This research was conducted at a university hospital in Gaziantep, South-east Turkey. The PICU is located in the pediatric hospital as separate department, only accessible to authorized staff. Age, gender, underlying diseases, treatment, and outcome were investigated from the patient files. The primary outcome measure for the patients with* E. meningoseptica*-positive clinical samples was discharge or death. 

### Microbiological method 

The standard method for routine identification and susceptibility testing was followed. All samples (patient and environmental surfaces) were inoculated onto Columbia agar (with 5% sheep blood) and eosin methylene blue Agar (BD, USA). The inoculated plates were incubated at 36°C in 5% CO2. After 24-h incubation, bacterial isolates were identified with matrix-assisted laser desorption/ionization time-of-flight mass spectrometry (MALDI-TOF MS, Bruker Daltonics Inc, USA). In addition, the identification and routine susceptibility testing of the isolates were performed using the Vitek^®^2 automated bacterial identification and antibiogram system (bioMérieux, France); a standard of 0.5 McFarland inoculum was prepared from the overnight colonies, and the suitable Vitek^®^ ID and AST cards were used according to the manufacturer’s recommendations. Standard procedures for blood cultures were followed. When the patient had a fever or when infection was suspected clinically, two sets of blood cultures were collected from different peripheral veins. Blood samples were inoculated into aerobic blood culture bottles and cultured using the BACTEC^®^ FX-400 system (Becton Dickinson, USA). 

Additionally, antimicrobial susceptibility testing (AST) was carried out using the Kirby-Bauer disk diffusion method according to recommendations of the European Committee on Antimicrobial Susceptibility Testing (EUCAST) [11] and the results were interpreted according to EUCAST guidelines [12]. Due to the absence of defined breakpoints for *E. meningoseptica*, results were interpreted in terms of the EUCAST zone diameter breakpoints for *Pseudomonas* spp. for ciprofloxacin, levofloxacin, piperacillin-tazobactam, amikacin, meropenem, ceftazidime, cefepime, tobramycin, and ceftazidime-avibactam; the EUCAST zone diameter breakpoints for *Enterococcus* spp. were applied for vancomycin; and the EUCAST zone diameter breakpoints for Enterobactereles were used for trimethoprim-sulfamethoxazole, gentamicin, and ceftaroline. Overnight bacterial cultures were adjusted to a turbidity equivalent to that of a 0.5 McFarland standard and spread over the entire surface, including the rim, of a Mueller Hinton Agar (MHA; BD, USA) medium using a sterile swab. A panel of 14 antibiotics was tested, employing the disk diffusion test. Antibiotic disks of vancomycin (10 µg), meropenem (10 µg), ciprofloxacin (5 µg), levofloxacin (5 µg), tobramycin (10 µg), piperacillin-tazobactam (36 µg), cefepime (30 µg), amikacin (30 µg), ceftazidime-avibactam (14 µg), gentamicin (10 µg), ampicillin (10 µg), ceftaroline (5 µg), amoxicillin-clavulanate (30 µg), and trimethoprim-sulfamethoxazole (25 µg) (all from Oxoid, UK) were placed on the inoculated MHA plates. The inoculated antibiogram plates were incubated at 36°C for 20 hours. The diameter of the inhibition zone was measured with a transparent ruler. Isolates were classified as susceptible (S), susceptible at increased exposure (I), areas of technical uncertainty (ATU), or resistant (R) according to the breakpoints defined by EUCAST 2021 [12]. Thereafter, antimicrobial susceptibility test results using the Kirby-Bauer method and Vitek^®^2 system were also compared.

### Outbreak source investigation 

After December 29, 2019, samples were collected from all potential surfaces and equipment, including ventilators, ventilator water reservoirs, endotracheal tubes, catheters, tap water, sink basins, faucets, doors and door handles, electrical buttons, telephones, computer keyboards, and covers of patient files, parenteral medication, infant formulas and antiseptic solutions. These samples were collected periodically each week. The environmental surveillance sampling started after the third isolation of *E. meningoseptica* on the PICU in January 2020 and ended in May 2020, when there had been no *E. meningoseptica*-positive cultures for one month. In total, 523 environmental samples were collected over the 5-month period. Sample cultures with Gram-negative bacillus, non-fermentative oxidase-positive colonies (n=82) were further tested for the presence of *E. meningoseptica*. Throat swabs and hand cultures were collected from 32 PICU staff members who had direct patient contact or equipment contact in the PICU, including physicians, nurses, and hospital cleaning staff. One set of cultures (throat swab and hand culture) were collected every week for 5 months.

### Infection control measures 

Patient admissions to the PICU were not restricted, but all patients with* E. meningoseptica*-positive samples were cohorted in a separate section on the PICU. Disposable aprons were used in this section. PICU access authorization was revised and limited. A general disinfection program using sodium hypochlorite solution was applied to all fomites. The infection control unit provided training to emphasize hand hygiene (washing hands with antimicrobial soap and alcohol rubbing) and contact precautions to reduce the risk of nosocomial transmission. 

## Results

### Environmental surveillance 

*E. meningoseptica* was isolated from 6 different patients in a total of 16 samples. These samples were taken from 8 tracheal aspirates (50%), 5 blood cultures (31%), 2 intravenous catheter tips (12%) and 1 cerebrospinal fluid (CSF) (6%) sample. Patient 1 had simultaneous positive cultures of catheter and peripheral blood. Positive signal time for catheter blood was 11 h 6 min and positive signal time for peripheral blood was 13 h 24 min. *E. meningoseptica* was isolated from 4 different environmental surfaces collected from two different ventilator water reservoirs, a ventilator device, and a computer keyboard. The screening of staff members did not reveal any positive (colonized) cases. 

### Patient characteristics 

During the 4.5-month outbreak, *E. meningoseptica* was isolated from 6 patients. Table 1 (Tab. 1) shows the clinical characteristics of these patients: ages ranged from 4 months to 11 months, with a median age of 9 months; 67% were males and 33% were females. In our patient group, the overall mortality rate was 66.6% (4 of 6), but patients also had severe underlying conditions worsened by nosocomial infections. All 4 deaths were observed in infants.

### Antibiotic susceptibility 

In our study, to our knowledge for the first time, the antimicrobial activity of recently developed antibiotics ceftazidime-avibactam and ceftaroline (as a fifth-generation cephalosporin) on *E. meningoseptica* were tested. Unfortunately, all isolates were resistant to both antibiotic agents in vitro. Subsequent positive cultures from the patients showed no difference in terms of antimicrobial susceptibility results (Table 2 (Tab. 2)).

## Discussion

To detect a common source of an *E. meningoseptica* outbreak, it is necessary to periodically collect samples from food and infant formulas, wet areas, dry surfaces, equipment, and the hands of healthcare workers [13]. The hospital water supply should be carefully monitored by the infection control units, and in case of any suspected source, necessary preventive actions should be taken in advance of pending microbiological results [14].

Regulating the protocol for empiric antibiotics, thorough disinfection of the unit, and restricting further admissions are also applied procedures during* E. meningoseptica* outbreaks in pediatric care centers [5], [7], [15]. However, other studies exist which describe effective outbreak control with less stringent measures, including the use of alcoholic hand rub after hand washing, care of infants with sterile instead of tap water, and repairing, cleaning, super-chlorinating, and isolating the water tanks from all hospital feeder tanks as well as changing the sink taps [5], [8], [16]. Discarding all opened materials such as creams, ointments, intravenous solutions, sterile water, hand-washing solutions, and infant formulas is also crucial, as these have also been reported as potential sources of the outbreak [2], [13], [17]. Especially in tertiary care centers in regions where there is no alternative specialized hospital, restriction of further admissions may not be a feasible strategy. The reported risk factors for *E. meningoseptica* infection include prolonged hospital stay, the presence of comorbidities, and the use of central venous catheters [18]. Most of the patients in our study had important underlying conditions, and *E. meningoseptica* was isolated from the central venous catheters of 2 patients.

At present, no consensus on first-line antimicrobial therapy for *E. meningoseptica* exists [18]. *E. meningoseptica* is resistant to many antimicrobial agents commonly used for nosocomial infections caused by Gram-negative bacteria, which often leads to ineffective treatment and high mortality rates following standard empirical therapy [3]. *E. meningoseptica* strains may carry genes encoding beta-lactamases, including extended-spectrum beta-lactamases and metallo-beta-lactamases. Therefore, they may display resistance to multiple antibiotics [19]. New antibiotic agents or new combinations are needed. Di Pentima et al. [20] stated that vancomycin and rifampin should be considered as the initial empirical therapy in newborns. Also, they suggested quinolones and oxazolidinones alone and in combination – as promising potential alternatives combined with vancomycin – they might also represent an alternative for improving clinical outcome [20]. In our study, all isolates were susceptible to ciprofloxacin and levofloxacin according to the Vitek^®^2-system results, but all isolates showed intermediate susceptibility (I) to ciprofloxacin and levofloxacin, as shown by the disk diffusion method. 

AST results for *E. meningoseptica* isolates are contradictory [21]. In a clinical study with 13 patients, all *E. meningoseptica* isolates were susceptible to piperacillin-tazobactam, and 78.6% of the isolates were susceptible to trimethoprim-sulfamethoxazole [22]. In our study, none of the isolates were susceptible to piperacillin-tazobactam and only 50% of the isolates were susceptible to trimethoprim-sulfamethoxazole according to automated Vitek^®^2 antimicrobial susceptibility test results. In addition, all isolates were resistant to trimethoprim-sulfamethoxazole, as shown by the disk diffusion method. Vitek^®^, an automated method of antimicrobial susceptibility testing, was shown to be less accurate than disk-diffusion susceptibility testing [23], [24]. In vitro AST results may not always predict the clinical outcome when infections with chryseobacteria are encountered; therefore, MICs may be no more valid than are E-test or disk diffusion results in testing these bacteria [25].

Vancomycin has been described as an active agent against *E. meningoseptica*, especially in cases of infantile meningitis due to *E. meningoseptica* [20]. However, conflicting results with high vancomycin MIC values against *E. meningoseptica* have also been reported [25], [26].

Chen et al. [27] stated that the survival rate of the patients who received cephalosporins, teicoplanin/vancomycin or piperacillin-tazobactam was highly variable. Delayed and inappropriate antibiotic therapy has been reported to be a have a significant negative impact on outcomes of patients with bacteremia [28]. In our study, 67% of the isolates were susceptible to vancomycin. For vancomycin, over 95% of the MICs determined by agar dilution and the E-test agreed within 1 log_2_ dilution of the results obtained by broth microdilution; there was only one major error with the disk diffusion method when enterococcal zone criteria was employed [25]. Our children received at least 4x15 mg/kg/day (60 mg/kg/day) vancoymcin.

*Elizabethkingia* species are an important cause of nosocomial infections, usually causing potentially avoidable hospital-acquired infections from sources such as water supplies or medical equipment. Most of the pediatric patients are immunocompromised due to intensive medical interventions, malnourishment, prematurity, or any of several other chronic or infectious conditions, and are vulnerable to opportunistic pathogens. Pediatric exposure to these bacteria creates an important risk for infection; therefore, stringent infection control protocols are crucial to avoid potential outbreak sources [29].

We identified some limitations in our study. Broth microdilution or agar microdilution is the reference method for AST, but in routine practice, it is highly labor intensive and it is not cost-effective to keep all antibiotic powders in stock (special forms of antimicrobial agents used in microdilution tests) which may be used in *E. meningoseptica* antimicrobial susceptibility testing.

## Conclusions

*E. meningoseptica* is a significant cause of nosocomial infections with high mortality especially in children. Investigations to identify the outbreak source and continuation of intensive infection control precautions are vital to handle *E. meningoseptica* outbreaks in PICUs. Optimal antimicrobial therapy guidelines for *E. meningoseptica* infections remain to be established. According to* in-vitro* test results derived from automated AST systems, using quinolones may cause inadequate treatment dosages and selection of resistant strains.

Infection control personnel in hospitals must be aware of *E. meningoseptica*, especially in long-stay patients in order to prevent transmission to the PICU. 

## Notes

### Competing interests

The authors declare that they have no competing interests.

### Acknowledgments 

Thanks to all PICU staff for their patience and cooperation. 

### Funding

Financial support was not granted for this study.

## Figures and Tables

**Table 1:**
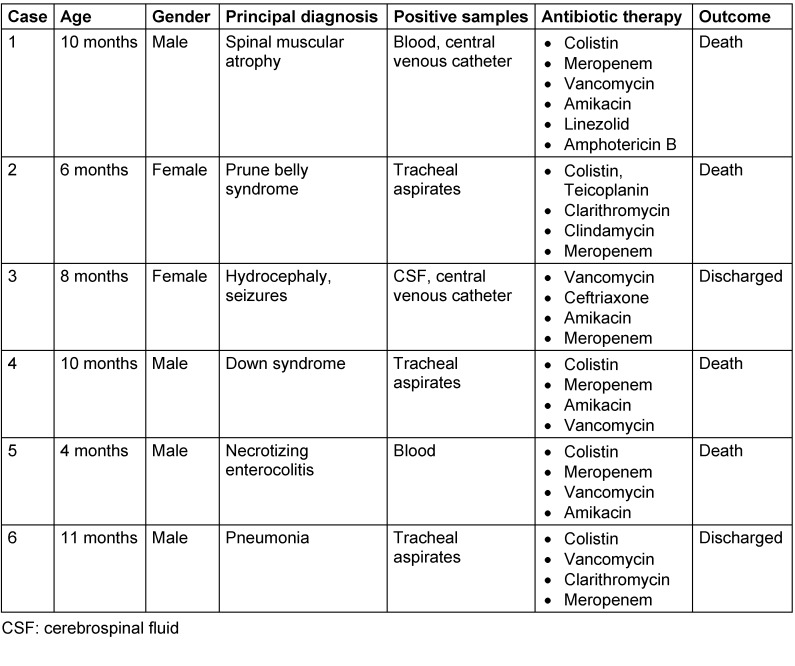
Clinical characteristics of patients with *E. meningoseptica* infection

**Table 2:**
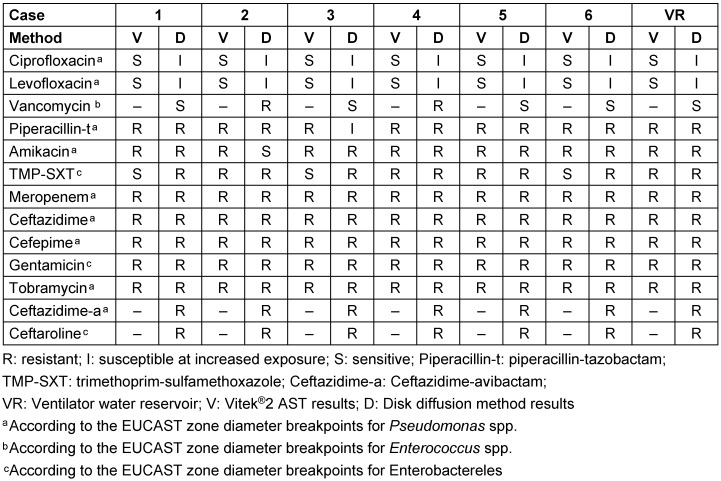
Antimicrobial susceptibility testing results

## References

[1] Hsu MS, Liao CH, Huang YT, Liu CY, Yang CJ, Kao KL, Hsueh PR (2011). Clinical features, antimicrobial susceptibilities, and outcomes of Elizabethkingia meningoseptica (Chryseobacterium meningosepticum) bacteremia at a medical center in Taiwan, 1999-2006. Eur J Clin Microbiol Infect Dis.

[2] Ceyhan M, Yildirim I, Tekeli A, Yurdakok M, Us E, Altun B, Kutluk T, Cengiz AB, Gurbuz V, Barin C, Bagdat A, Cetinkaya D, Gur D, Tuncel O (2008). A Chryseobacterium meningosepticum outbreak observed in 3 clusters involving both neonatal and non-neonatal pediatric patients. Am J Infect Control.

[3] Jean SS, Lee WS, Chen FL, Ou TY, Hsueh PR (2014). Elizabethkingia meningoseptica: an important emerging pathogen causing healthcare-associated infections. J Hosp Infect.

[4] Brooks GF, Carroll KC, Butel JS, Morse SA (2010). Medical Microbiology.

[5] Güngör S, Ozen M, Akinci A, Durmaz R (2003). A Chryseobacterium meningosepticum outbreak in a neonatal ward. Infect Control Hosp Epidemiol.

[6] du Moulin GC (1979). Airway colonization by Flavobacterium in an intensive care unit. J Clin Microbiol.

[7] Hoque SN, Graham J, Kaufmann ME, Tabaqchali S (2001). Chryseobacterium (Flavobacterium) meningosepticum outbreak associated with colonization of water taps in a neonatal intensive care unit. J Hosp Infect.

[8] Tekerekoglu MS, Durmaz R, Ayan M, Cizmeci Z, Akinci A (2003). Analysis of an outbreak due to Chryseobacterium meningosepticum in a neonatal intensive care unit. New Microbiol.

[9] Nulens E, Bussels B, Bols A, Gordts B, Van Landuyt HW (2001). Recurrent bacteremia by Chryseobacterium indologenes in an oncology patient with a totally implanted intravascular device. Clin Microbiol Infect.

[10] Jean SS, Hsieh TC, Ning YZ, Hsueh PR (2017). Role of vancomycin in the treatment of bacteraemia and meningitis caused by Elizabethkingia meningoseptica. Int J Antimicrob Agents.

[11] Matuschek E, Brown DF, Kahlmeter G (2014). Development of the EUCAST disk diffusion antimicrobial susceptibility testing method and its implementation in routine microbiology laboratories. Clin Microbiol Infect.

[12] The European Committee on Antimicrobial Susceptibility Testing (EUCAST) (2021). Breakpoint tables for interpretation of MICs and zone diameters.

[13] Steinberg JP, Mandell GL, Bennett JE, Dolin R (2000). Other gram-negative bacilli. Principles and Practice of Infectious Diseases.

[14] Ceyhan M, Celik M (2011). Elizabethkingia meningosepticum (Chryseobacterium meningosepticum) Infections in Children. Int J Pediatr.

[15] Hazuka BT, Dajani AS, Talbot K, Keen BM (1977). Two outbreaks of Flavobacterium meningosepticum type E in a neonatal intensive care unit. J Clin Microbiol.

[16] Abrahamsen TG, Finne PH, Lingaas E (1989). Flavobacterium meningosepticum infections in a neonatal intensive care unit. Acta Paediatr Scand.

[17] Adachi A, Mori T, Shimizu T, Yokoyama A, Takayama N, Ikeda Y, Okamoto S (2004). Chryseobacterium meningosepticum septicemia in a recipient of allogeneic cord blood transplantation. Scand J Infect Dis.

[18] Rastogi N, Mathur P, Bindra A, Goyal K, Sokhal N, Kumar S, Sagar S, Aggarwal R, Soni KD, Tandon V (2016). Infections due to Elizabethkingia meningoseptica in critically injured trauma patients: a seven-year study. J Hosp Infect.

[19] Vessillier S, Docquier JD, Rival S, Frere JM, Galleni M, Amicosante G, Rossolini GM, Franceschini N (2002). Overproduction and biochemical characterization of the Chryseobacterium meningosepticum BlaB metallo-beta-lactamase. Antimicrob Agents Chemother.

[20] Di Pentima MC, Mason EO, Kaplan SL (1998). In vitro antibiotic synergy against Flavobacterium meningosepticum: implications for therapeutic options. Clin Infect Dis.

[21] Kirby JT, Sader HS, Walsh TR, Jones RN (2004). Antimicrobial susceptibility and epidemiology of a worldwide collection of Chryseobacterium spp: report from the SENTRY Antimicrobial Surveillance Program (1997-2001). J Clin Microbiol.

[22] Chan JC, Chong CY, Thoon KC, Tee NWS, Maiwald M, Lam JCM, Bhattacharya R, Chandran S, Yung CF, Tan NWH (2019). Invasive paediatric Elizabethkingia meningoseptica infections are best treated with a combination of piperacillin/tazobactam and trimethoprim/sulfamethoxazole or fluoroquinolone. J Med Microbiol.

[23] Bolash NK, Liu HH (1995). Quinolone susceptibility of multiply-resistant Flavobacterium meningosepticum clinical isolates in one urban hospital. Drugs.

[24] Sader HS, Jones RN, Pfaller MA (1995). Relapse of catheter-related Flavobacterium meningosepticum bacteremia demonstrated by DNA macrorestriction analysis. Clin Infect Dis.

[25] Fraser SL, Jorgensen JH (1997). Reappraisal of the antimicrobial susceptibilities of Chryseobacterium and Flavobacterium species and methods for reliable susceptibility testing. Antimicrob Agents Chemother.

[26] Chang JC, Hsueh PR, Wu JJ, Ho SW, Hsieh WC, Luh KT (1997). Antimicrobial susceptibility of flavobacteria as determined by agar dilution and disk diffusion methods. Antimicrob Agents Chemother.

[27] Chen WC, Chen YW, Ko HK, Yu WK, Yang KY (2020). Comparisons of clinical features and outcomes between Elizabethkingia meningoseptica and other glucose non-fermenting Gram-negative bacilli bacteremia in adult ICU patients. J Microbiol Immunol Infect.

[28] Ibrahim EH, Sherman G, Ward S, Fraser VJ, Kollef MH (2000). The influence of inadequate antimicrobial treatment of bloodstream infections on patient outcomes in the ICU setting. Chest.

[29] Dziuban EJ, Franks JL, So M, Peacock G, Blaney DD (2018). Elizabethkingia in Children: A Comprehensive Review of Symptomatic Cases Reported From 1944 to 2017. Clin Infect Dis.

